# A Descriptive Observational Study of Real-Time TDM-Guided Aggressive PK/PD Target Attainment and Microbiological/Clinical Outcome in Patients with *Stenotrophomonas maltophilia* Bloodstream Infections and/or Pneumonia Treated with Continuous Infusion Cefiderocol

**DOI:** 10.3390/antibiotics15100990

**Published:** 2026-10-07

**Authors:** Milo Gatti, Matteo Rinaldi, Simone Ambretti, Maddalena Giannella, Pierluigi Viale, Federico Pea

**Affiliations:** 1 Department of Medical and Surgical Sciences, Alma Mater Studiorum University of Bologna, 40138 Bologna, Italy; simone.ambretti3@unibo.it (S.A.); maddalena.giannella@unibo.it (M.G.); pierluigi.viale@unibo.it (P.V.); federico.pea@unibo.it (F.P.); 2 Clinical Pharmacology Unit, Department for Integrated Infectious Risk Management, IRCCS Azienda Ospedaliero-Universitaria di Bologna, 40138 Bologna, Italy; 3 Infectious Disease Unit, Department for Integrated Infectious Risk Management, IRCCS Azienda Ospedaliero-Universitaria of Bologna, 40138 Bologna, Italy; matteo.rinaldi@aosp.bo.it; 4 Operative Unit of Microbiology, Department for Integrated Infectious Risk Management, IRCCS Azienda Ospedaliero-Universitaria di Bologna, 40138 Bologna, Italy

**Keywords:** cefiderocol, continuous infusion, *Stenotrophomonas maltophilia*, TDM-guided strategy, bloodstream infection, pneumonia, clinical outcome, microbiological failure

## Abstract

Objectives: To assess whether attaining an aggressive pharmacokinetic/pharmacodynamic (PK/PD) target with continuous infusion (CI) cefiderocol could be an effective strategy for managing documented bloodstream infections (BSIs) and pneumonia caused by *Stenotrophomonas maltophilia*, and to identify independent predictors of microbiological and clinical failure. Methods: Patients receiving CI cefiderocol according to a real-time therapeutic drug monitoring (TDM)-guided expert clinical pharmacological advice (ECPA) program for managing documented BSIs and/or pneumonia caused by *Stenotrophomonas maltophilia* were retrospectively included. Free fractions of plasma cefiderocol steady-state concentrations (*f*C_ss_) were calculated according to a protein binding of 58%, and aggressive PK/PD target was defined as the attainment of a *f*C_ss_/MIC ratio > 4. Exploratory multivariate analysis was implemented for assessing potential independent predictors of microbiological and clinical failure. Results: Overall, 25 patients (14 BSIs, 10 pneumonia, and one bacteremic pneumonia) were included. Early and overall aggressive cefiderocol PK/PD target was attained in 100.0% of cases. Microbiological eradication rate was 75.0% (15/20) among patients having available follow-up cultures, whereas clinical cure rate was 60.0% (15/25). No case of resistance occurrence to cefiderocol emerged. Bacteremia emerged as the only potential predictor of reduced risk of microbiological failure (odds ratio [OR] 0.04; 95%CI 0.01–0.54; *p* = 0.016), whereas mechanical ventilation was potentially associated with higher risk of clinical failure (OR 15.17; 95%CI 2.03–111.35; *p* = 0.008). No significant differences between cefiderocol mono- or combination therapy in terms of microbiological and/clinical outcome were found. Conclusions: Our findings could suggest that CI cefiderocol could be a feasible way for attaining aggressive PK/PD target. This approach could represent a valuable strategy for favoring the microbiological eradication and/or clinical cure of documented *Stenotrophomonas maltophilia* infections with cefiderocol monotherapy in critical immunocompromised patients. Prospective comparative studies would be warranted for confirming our hypothesis.

## 1. Introduction

*Stenotrophomonas maltophilia* is an environmental global emerging multidrug-resistant Gram-negative pathogen causing several types of hospital-acquired infections, especially among immunocompromised patients [1]. Although this pathogen has usually low virulence, and distinguishing between colonization or true infection could be challenging [2], the crude mortality rate of *Stenotrophomonas maltophilia* hospital-acquired infections may be remarkable, ranging from 14% to 69% for pneumonia and bloodstream infections (BSIs) [3,4]. Additionally, a peculiar feature of this pathogen is the capability of producing biofilm on the surface of different types of devices, thus representing an important potential etiological agent of catheter-related BSIs (CR-BSIs) [1,5,6].

The intrinsic multidrug resistance of *Stenotrophomonas maltophilia* makes treating severe infections very challenging [7]. Historically, cotrimoxazole was considered as first-line therapy based especially on its good in vitro activity, and some favorable clinical outcomes have been observed in real-world evidence [7]. Alternatively, both levofloxacin and minocycline has been considered potentially suitable for this purpose [7]. However, treating severe *Stenotrophomonas maltophilia* infections with a monotherapy based on one of the aforementioned agents is currently arguable and a matter of debate. On the one hand, concerns have emerged in defining appropriate clinical breakpoints against this pathogen, and, on the other hand, no strong correlation between pharmacokinetic/pharmacodynamic (PK/PD) target attainment and clinical outcome have been established yet [7]. Accordingly, the last updated version of the Infectious Diseases Society of America (IDSA) guidance recommends using these agents in combination when treating *Stenotrophomonas maltophilia* infections [8].

Cefiderocol, a recently licensed siderophore cephalosporin, was characterized by excellent in vitro activity against *Stenotrophomonas maltophilia* [9]. Interestingly, two recent systematic reviews with meta-analyses reported that the resistance rate to cefiderocol among *Stenotrophomonas maltophilia* isolates was very low, namely 0.4–4% [10,11]. Nonetheless, in both the CREDIBLE-CR [12] and the GAME CHANGER pivotal trials [13], some unfavorable clinical outcomes were reported in treating *Stenotrophomonas maltophilia* infections. Regardless, several preclinical models [14,15,16] and some real-world clinical data [17,18,19,20] may support the potential efficacy of cefiderocol in this setting. Based on this, the last updated version of the IDSA guidance suggested cefiderocol monotherapy as being preferred for treating documented *Stenotrophomonas maltophilia* infections [8], despite randomized comparative trials being currently lacking [21].

Cefiderocol, and likewise, the other beta-lactams, exhibits time-dependent antibacterial activity, since the best pharmacodynamic target of efficacy is represented by the time with free concentrations persisting above the minimum inhibitory concentrations (%*f*T_>MIC_) [22]. In recent years, an ever-growing number of studies supported the relevance of attaining an aggressive PK/PD target of 100%*f*T_>4×MIC_ with beta-lactams, including cefiderocol, when treating severe infections in the critically ill patients for both maximizing clinical outcome and microbiological eradication, and for preventing resistance development against Gram-negative pathogens [23,24,25,26]. A recent meta-analysis of 21 studies concerning the treatment of Gram-negative infections in critically ill patients showed that administering beta-lactams by prolonged or continuous infusion (CI) may significantly decrease the risk of early aggressive PK/PD target non-attainment [24]. Additionally, another meta-analysis of 11 studies assessing the role of beta-lactam antibiotic therapeutic drug monitoring (TDM) in critically ill patients showed that a TDM-guided strategy significantly improved both clinical and microbiological outcomes other than optimal PK/PD target attainment [27].

Based on these rationales, over several years, delivering beta-lactams by CI and optimizing aggressive PK/PD target attainment by means of a real-time TDM-guided strategy has become standard practice at our hospital for maximizing the treatment of severe Gram-negative infections with beta-lactams, including cefiderocol, in critically ill patients [25].

The aim of this retrospective study was to describe the relationship between the TDM-guided aggressive PK/PD target attainment of CI cefiderocol and microbiological/clinical outcome in patients with documented BSIs and pneumonia caused by *Stenotrophomonas maltophilia*, and to identify the independent predictors of microbiological and clinical failure.

## 2. Results

Overall, 25 patients received targeted therapy with real-time TDM-guided CI cefiderocol for the management of documented BSIs or pneumonia caused by *Stenotrophomonas maltophilia*. Demographics and clinical features of the included patients are reported in Table 1.

The median (IQR) age was 63 years (40–67 years), with a male preponderance (72.0%). The median (IQR) Charlson Comorbidity Index (CCI) was 5 (3–7). Hematological malignancies (11 cases; 44.0%) were the most frequent underlying disease, followed by solid organ transplantation (6 cases; 24.0%), and solid cancer (3 cases; 12.0%). A total of 88.0% (22 cases) of patients were immunosuppressed.

Patients were mostly admitted to the intensive care unit (15 cases; 60.0%), followed by hematology and medical ward (5 cases each; 20.0%). Vasopressors support and mechanical ventilation were required in 10 (40.0%) and 9 patients (36.0%), respectively. Continuous renal replacement therapy (CRRT) was implemented in seven cases (28.0%), one of which was associated with the use of a hemoadsorptive filter. Augmented renal clearance (ARC) occurred in only one patient. Hypoalbuminemia was reported in 19 cases (76.0%).

Hospital-acquired (HAP) and ventilator-associated pneumonia (VAP) were the most prevalent types of infection (10 cases; 40.0%), followed by CR-BSI (9 cases; 36.0%), secondary BSI (3 cases; 12.0%), primary BSI (2 cases; 8.0%), and VAP plus BSI (1 case; 4.0%). All of the *Stenotrophomonas maltophilia* clinical isolates were susceptible to cefiderocol at baseline, with a median (IQR) MIC of 0.125 mg/L (0.06–2 mg/L).

Cefiderocol by CI was administered at a median (IQR) daily maintenance dose (MD) of 6 g (3–6 g). Median (IQR) treatment duration was 8 days (5–14 days). Cefiderocol was used as a monotherapy in 14 cases (56%) and as combination therapy in 11 cases (44.0%) [with cotrimoxazole (n = 6), levofloxacin (n = 4), and minocycline (n = 1)]. Source control was feasible in 12 out of 25 included cases, being complete and effective in 10 out of these (83.3%). Effective source control was not performed in the two remaining cases having BSI with a probable abdominal source.

Overall, 48 real-time TDM-guided expert clinical pharmacological advice (ECPA) were provided for optimizing CI cefiderocol exposure, accounting for a median (IQR) of 1 (1–3) per patient. Median (IQR) and range cefiderocol *f*C_ss_/MIC ratio were 21.6 (12.2–87.3) and 7.8–914.8, respectively. All of the 25 included patients attained aggressive PK/PD target, both early and overall. Dosing reduction was applied on 27 out of 48 real-time TDM-guided ECPAs (56.2%), whereas an increase was recommended only in one ECPA (2.1%) (namely in a patient undergoing CRRT equipped with oXiris hemoadsorptive filter).

Clinical cure was reported in 60.0% of included patients (15/25). Follow-up microbiological cultures were available in 20 out of 25 included cases. Microbiological eradication was obtained in 75.0% of assessable patients (15/20). No case of 90-day resistance development emerged in the 14 patients having assessable microbiological outcome and alive at 90 day. The 30-day mortality rate was 40.0% (10/25).

Univariate and multivariate analyses assessing the potential predictors of microbiological eradication among the 20 patients having follow-up microbiological cultures are summarized in Table 2.

At multivariate analysis, overall bacteremia emerged as the only potential predictor of reduced risk of microbiological failure (OR 0.04; 95%CI 0.01–0.54; *p* = 0.016).

Univariate and multivariate analyses assessing potential predictors of clinical failure are reported in Table 3.

During multivariate analysis, mechanical ventilation emerged as the only potential predictor of clinical failure (OR 15.17; 95%CI 2.03–111.35; *p* = 0.008).

Microbiological and clinical outcomes based on the site of infection and mono- or combination therapy are detailed in Figure 1.

## 3. Discussion

To the best of our knowledge, this is the first retrospective case series investigating the impact of an aggressive PK/PD target attainment based on TDM-guided CI cefiderocol in the treatment of documented infections caused by *Stenotrophomonas maltophilia,* and is also the largest one evaluating its clinical efficacy in this challenging scenario.

Overall, the findings showed that this strategy may allow one to attain aggressive PK/PD target both early and overall in all of the included cases. This approach led to both good microbiological eradication rate (75.0%) and clinical cure rate (60.0%), with no case of resistance emergence. Interestingly, bacteremia resulted in being protective against microbiological failure, whereas mechanical ventilation increased the risk of clinical failure.

The recent 2026 IDSA guidance suggests using cefiderocol as monotherapy for the first-line treatment of *Stenotrophomonas maltophilia* infections, based mainly on data coming from preclinical models [8]. In fact, clinical evidence supporting its role in this challenging scenario is quite limited and conflicting [12,13,17,18,20]. On the one hand, the findings of the two pivotal trials were quite disappointing on this issue. Both the CREDIBLE-CR trial [12] and the GAME CHANGER trial [13] reported a high mortality rate among the few included patients having *Stenotrophomonas maltophilia* infections treated with cefiderocol (80.0%, 4/5 and 40.0%, 2/5, respectively). On the other hand, real-world evidence showed promising findings [17,18,20]. A subgroup analysis of 20 patients having *Stenotrophomonas maltophilia* infections (55.0% pneumonia) treated with cefiderocol in the multicenter retrospective observational PERSEUS study reported a clinical cure and mortality rate of 70.0% and 30.0%, respectively [17]. Similarly, a series of eight cases of *Stenotrophomonas maltophilia* infections (5 CR-BSIs, 2 pneumonia, and 1 skin and soft tissue infection) showed clinical cure rate and microbiological eradication rate with cefiderocol of 62.5% and 87.5%, respectively, with an in-hospital mortality of 37.5% [18]. A retrospective case series including 10 patients with hematological malignancies having documented *Stenotrophomonas maltophilia* infections (60% pneumonia and 30% CR-BSI) treated with cefiderocol reported a high rate of clinical improvement (90.0%), with quite a remarkable mortality rate both at 30 day (30.0%) and in hospital (50.0%) [20].

Our findings are in agreement with these latter observational studies. Regarding the quite high 30-day mortality rate, it should be noticed that, in our cohort, approximately 90% of patients were immunosuppressed, and half had hematological malignancies as underlying diseases. This is consistent with the available evidence showing a significantly increased risk of *Stenotrophomonas maltophilia* infections among immunocompromised and hematological patients [20,28,29,30], with neutropenia and malignancy being independent predictors of mortality [31,32].

Our approach could add important knowledge to the current literature. Aggressive beta-lactams’ PK/PD target attainment represents a strategy that has been recently shown to be extremely helpful in maximizing both clinical outcome and microbiological eradication, and especially in suppressing resistance development when treating Gram-negative infections in critically ill patients [24]. Interestingly, in our cohort, delivering cefiderocol by CI allowed the early attaining of an aggressive PK/PD target in all of the included cases. In this regard, we showed, in a recent retrospective study assessing the utility of a TDM-guided ECPA program for optimizing aggressive PK/PD target attainment with novel beta-lactams, that the likelihood of aggressive PK/PD target attainment with cefiderocol was much higher when delivering by CI rather than by the standard 3 h extended infusion (EI) (97.1 vs. 73.5%), and that the former mode was associated with the highest proportion of clinical cure rate (50.0% of assessable clinical outcomes; *p* = 0.006) [25]. Unfortunately, in the present study, the attainment of an aggressive PK/PD target in all included patients precluded us from evaluating its impact on outcome. However, this may be indirectly inferred by the findings of a retrospective case series of PK/PD target attainment and microbiological outcome among 13 critically ill patients having pneumonia or BSI due to carbapenem-resistant *Acinetobacter baumannii* being treated with cefiderocol, mostly as monotherapy administered by standard EI [26]. Microbiological failure occurred in as much as 55.5% of patients having aggressive PK/PD target non-attainment, but in only 25% of those having an aggressive PK/PD target attainment [26].

The TDM-guided approach was especially helpful in allowing confirmation of aggressive PK/PD target attainment even by decreasing the dosages in almost 50% of cases. This was enabled by the good in vitro susceptibility of most of the *Stenotrophomonas maltophilia* clinical isolates and was valuable in decreasing the risk of selective pressure associated with clinically not useful high-antibiotic-dosing regimens leading to potential resistance development. Accordingly, this approach could be protective against 90-day resistance development to cefiderocol since no case occurred in our series. Indeed, this should be confirmed in larger prospective studies since only around half of the included patients was assessable for this outcome in the absence of a comparator group. Implementing a dedicated Bayesian model could provide additional benefits in guiding properly cefiderocol dosing adjustments under different pathophysiological conditions. This approach may allow one to estimate individual pharmacokinetic parameters in each patient, and could represent a relevant topic for future research.

Bacteremia emerged as the only potential predictor of microbiological eradication. This is not surprising and can be explained by two different aspects. On the one hand, aggressive PK/PD target attainment at the infection site may be easier for cefiderocol, and likewise for other hydrophilic beta-lactams, in treating BSI than pneumonia, since approximately only 30% of plasma concentrations may penetrate the epithelial lining fluid [33]. On the other hand, effective source control, namely a mandatory issue in the management of Gram-negative infections [34], may be easily implemented in BSI, especially in the case of CR-BSI, namely the most frequent type of BSI occurring in our cohort, simply by removing the catheter. This is consistent with previous studies showing that, in CR-BSI, due to *Stenotrophomonas maltophilia,* catheter removal was significantly associated with better clinical outcome [6,31].

Mechanical ventilation emerged as the only potential predictor of clinical failure. This may be explained by the fact that mechanical ventilation may be an independent risk factor for acquiring *Stenotrophomonas maltophilia* pneumonia in the intensive care unit (ICU) setting, and may favor the formation of *S. maltophilia*-embedding biofilm [35,36].

However, the predictors of microbiological/clinical outcome identified in our study should be interpreted cautiously given the small sample size and the low number of outcome events, and the multivariate regression should be considered as an exploratory analysis.

Importantly, we reported, as a hypothesis-generating observation, that attaining the aggressive PK/PD target of CI cefiderocol could maximize monotherapy efficacy since combination therapy with other anti-*S. maltophilia* active agents did not result in improving either clinical outcome or microbiological eradication. This could concur in shedding some light on the clinical setting for further supporting the recommendations provided by the last updated IDSA guidance stating that the data for supporting improved clinical outcomes with the use of cefiderocol as part of a combination therapy are insufficient [8]. However, the findings should be interpreted cautiously considering that the use of combination therapy could be subjected to confounding by indication.

Our study has several limitations. The retrospective observational study design, the limited sample size, and the lack of a control group (namely patients receiving standard dosing regimens of cefiderocol delivered by extended infusion in the absence of a TDM-guided strategy) should be recognized. Attainment of the aggressive cefiderocol PK/PD target in all of the included patients of our cohort precluded us from testing its real impact on outcome. The reliability of the multivariate regression analysis might have been potentially hampered by the small sample size and the low number of outcome events, which could have potentially increased the risk of model overfitting and unstable estimates. Larger confirmatory prospective studies would be warranted for identifying the definite independent predictors of microbiological/clinical outcome. Only total cefiderocol concentrations were measured, and the free fractions were calculated based on the plasma protein binding reported in the literature without considering any potential occurrence of hypoalbuminemia and/or of any other pathophysiological variation possibly affecting aggressive PK/PD target attainment. Follow-up microbiological cultures were unavailable in 20% of cases, introducing a possible ascertainment bias. Consequently, data on microbiological outcome should be interpreted cautiously. Calculating average cefiderocol *f*C_ss_ before and after dosing adjustments could have potentially masked periods of under- and/or overexposure, even if underexposure never emerged at each real-time TDM assessment. Finally, the use of combination therapy could be subjected to confounding by indication, considering that this choice could be influenced by disease severity, the presence of deep-seated infection and/or clinical judgment. Conversely, the very homogeneous cohort of patients, and the assessment of PK/PD target attainment may represent the points of strength of our study.

In conclusion, our findings could suggest that a real-time TDM-guided ECPA program could be a feasible way for attaining the aggressive PK/PD target with CI cefiderocol, and could be associated with the microbiological eradication and/or clinical cure of documented *Stenotrophomonas maltophilia* infections with cefiderocol monotherapy in critical immunocompromised patients. Prospective comparative studies, including cases failing in aggressive PK/PD target attainment with CI cefiderocol and a control group receiving standard dosing regimens of cefiderocol in the absence of a TDM-guided strategy, would be warranted for confirming our hypothesis. This approach could allow a better assessment of the potential advantages associated with cefiderocol monotherapy versus the other anti-*Stenotrophomonas* agents in this challenging scenario.

## 4. Materials and Methods

### 4.1. Study Design

We performed a retrospective, monocentric, observational study including patients hospitalized in the period of 1 January 2022–31 March 2026 at the IRCCS Azienda Ospedaliero-Universitaria, Bologna, Italy, who underwent the real-time TDM-guided strategy of CI cefiderocol for optimizing PK/PD target attainment in the management of documented BSI and/or pneumonia caused by *Stenotrophomonas maltophilia*. The intent was to evaluate the relationship between the aggressive PK/PD target attainment of CI cefiderocol and clinical/microbiological outcome in the challenging and poorly investigated setting of *Stenotrophomonas maltophilia* infections. A small proportion of patients included here (i.e., 28%) were also included in a previous study assessing methodologically the utility of the TDM-guided ECPA program in optimizing over-time aggressive PK/PD target attainment with novel beta-lactams [25]. The study was conducted according to the guidelines of the Declaration of Helsinki and was approved by the Area Vasta Emilia Centro Ethical Committee (CE-AVEC; No. 308/2021/Oss/AOUBo on 17 May 2021, EM232–2022_308/2021/Oss/AOUBo on 16 March 2022, and EM449–2023_308/2021/Oss/AOUBo on 17 April 2024). Informed written consent was waived due to the retrospective and observational nature of the study, according to the article 110-bis comma 4 of Legislative Decree No. 196/2003 rev. 2024 of Italian Law regulating retrospective study.

### 4.2. Data Collection and Outcome Definition

Demographic [age, sex, weight, height, and body mass index (BMI)], clinical/laboratory [CCI, underlying diseases, admission ward, presence of immunosuppression, baseline creatinine clearance (CLCr), requirement for mechanical ventilation and/or vasopressors, need of CRRT or of intermittent hemodialysis (IHD), occurrence of ARC, serum albumin], microbiological (type of infection, precise MIC values of *Stenotrophomonas maltophilia* clinical isolates), cefiderocol treatment (daily maintenance dosing regimen, treatment duration, mono- or combination therapy, overall TDM assessments and number of TDM per patient, number of TDM above, below, or within the desired therapeutic range and associated dosing adjustments, plasma steady-state cefiderocol concentrations, aggressive PK/PD target attainment or non-attainment at first and subsequent TDM assessments, implementation of an effective source control), and outcome data (microbiological eradication/failure, 90-day resistance development, clinical cure/failure, and 30-day mortality rate) were collected in each included case.

Obesity was defined as a BMI > 30 kg/m^2^ [37,38]. Immunosuppression was defined as the presence of at least one of the following conditions: long-term treatment with corticosteroids and/or biologic and/or antineoplastic agents, solid or hematologic malignancies, solid organ or hematopoietic stem cell transplantation, HIV disease or autoimmune disease [39].

CLCr was estimated by means of the CKD-EPI formula [40]. ARC was defined as a normal serum creatinine level coupled with an estimated CLCr > 130 mL/min/1.73 m^2^ in males and >120 mL/min/1.73 m^2^ in females [41].

Hypoalbuminemia was defined as a serum albumin level < 3.5 g/dL.

Types of infection were defined according to the Centers for Disease Control and Prevention (CDC) criteria [42,43]. Specifically, BSI was defined as the isolation of *Stenotrophomonas maltophilia* from at least one blood culture [42,43]. CR-BSI was defined as the isolation of *Stenotrophomonas maltophilia* from blood culture drawn from the device more than two hours before performing blood culture from venipuncture [42]. HAP was defined as the isolation of *Stenotrophomonas maltophilia* from the endotracheal aspirate culture with a bacterial load ≥10^6^ CFU/mL after more than 48 h of hospital admission [42]. VAP was defined as the isolation of *Stenotrophomonas maltophilia* from the bronchoalveolar lavage fluid culture with a bacterial load of ≥10^4^ CFU/mL after more than 48 h of endotracheal intubation and the start of mechanical ventilation [42].

Source control was considered a mandatory step for properly addressing the issue of some specific types of infection, and was defined as effective in the case of device removal for CR-BSI, or abscess drainage and/or surgical approach in the case of secondary BSI having an intrabdominal or cutaneous/subcutaneous source. Conversely, source control was defined as unfeasible in the case of primary BSI or pneumonia (HAP/VAP).

Microbiological eradication was defined as the absence of the index *Stenotrophomonas maltophilia* isolate at the infection site after at least 7 days of treatment [44]. Microbiological failure was conversely defined as the persistence of the index *Stenotrophomonas maltophilia* isolate at the site of infection, detected at follow-up microbiological cultures [44]. In the case of an absence of follow-up cultures, microbiological outcome was unassessable. Resistance development to cefiderocol was defined as an increase in the MIC value of the index *Stenotrophomonas maltophilia* isolate above the non-species-specific EUCAST clinical breakpoint of 2 mg/L within 90 days from starting of treatment [45]. Clinical cure was defined as the complete resolution of signs and symptoms of infection associated with documented microbiological eradication at the end of treatment, and the absence of either recurrence/relapse at 30-day follow-up or attributable mortality due to *Stenotrophomonas maltophilia*-related infection [46].

### 4.3. Antimicrobial Susceptibility Testing for Cefiderocol

Cefiderocol susceptibility was tested according to the current EUCAST recommendations [47,48]. First-level testing was performed by disk diffusion (30-µg cefiderocol disk) on standard Mueller–Hinton agar following the EUCAST standardized methodology. For *Stenotrophomonas maltophilia*, disk diffusion is considered an appropriate routine screening method because of its good correlation with the reference broth microdilution (BMD) method [49]. BMD was performed as second-level testing only for isolates with zone diameters within the EUCAST Area of Technical Uncertainty (ATU) or whenever confirmation of cefiderocol activity was clinically required. Reference MIC testing was carried out using iron-depleted cation-adjusted Mueller–Hinton broth (ID-CAMHB), as recommended by EUCAST, since conventional CAMHB may produce unreliable cefiderocol MIC values. This approach combines the easiness of use of routine disk diffusion with the higher accuracy of reference MIC determination for isolates with uncertain results, ensuring a reliable categorization of susceptibility in patients having severe infections like bloodstream infection or pneumonia caused by *Stenotrophomonas maltophilia*.

### 4.4. Cefiderocol Dosing Regimen, Sampling Procedure, and Optimization of Aggressive PK/PD Target Attainment

Cefiderocol treatment was always started with a loading dose (LD) of 2 g over 3 h, immediately followed by an initial MD administered by CI, namely q8h, q12h, or q24h over 8 h, 12 h, or 24 h infusions, according to a documented stability up to 24 h in polypropylene syringes [50]. The MD was selected on a case-by-case basis, taking into account the underlying pathophysiological conditions and the renal function of the patients. In patients undergoing CRRT or having sepsis-associated acute kidney injury, a full initial MD was administered to counteract the potential risk of having underexposure in the first 24–48 h of treatment [51,52].

Cefiderocol dosing regimens were subsequently adjusted by means of a real-time TDM-guided ECPA program. For this purpose, TDM of cefiderocol was first assessed after at least 24 h from starting therapy, and subsequently reassessed every 48–72 h whenever feasible. Total cefiderocol steady-state plasma concentrations (C_ss_) were measured by means of validated liquid chromatography–tandem mass spectrometry methods [53].

Real-time TDM-guided ECPAs were performed by well-trained MD clinical pharmacologists according to the “antimicrobial puzzle” concepts and made available to clinicians within 24 h, as previously detailed [54]. Specifically, dosing regimens were adjusted in order to attain an aggressive PK/PD target, defined as a cefiderocol-free C_ss_-to-MIC ratio (*f*C_ss_/MIC) > 4 (equivalent to 100% *f*T_>4×MIC_) [25]. The rationale for selecting this aggressive PK/PD target resulted from the preclinical and clinical evidence supporting its association with the improvement in clinical efficacy coupled with the suppression of resistance development in the scenario of Gram-negative infections [23,24,55]. Cefiderocol dosing adjustments were adopted as previously detailed [25]. Specifically, a 25% or 50% dosing reduction was performed whenever the *f*C_ss_/MIC ratio ranged between 8 and 10, or was >10, respectively. Conversely, a 25% or 50% dosing increase was adopted whenever the *f*C_ss_/MIC ratio ranged between 2 and 4, or was <2, respectively. In the case of a *f*C_ss_/MIC ratio ranging between 4 and 8, no adjustment was implemented and the current dosing regimen was confirmed [25]. These ranges should be considered as a rough guide as previously reported [25,56,57]. Dosing adjustments were performed on a case-by-case basis by means of an expert interpretation of TDM results according to the so-called “antimicrobial puzzle” concepts. This approach takes into account the site of infection and penetration rate of cefiderocol in the epithelial lining fluid according to the literature data, the precise MIC value of the clinical isolate, and the different pathophysiological alterations fluctuating over time (particularly in regard to ARC occurrence, CRRT initiation or withdrawal, and type of adopted membrane filter).

The cefiderocol-free fraction (*f*) was calculated by subtracting from the total measured C_ss_ the specific protein bound rate reported in the literature, and equal to 58% [58]. Specifically, the following equation was adopted for calculating the cefiderocol-free fraction (*f*): *f*C_ss_ = total cefiderocol C_ss_ * 0.42. The attainment of a cefiderocol aggressive PK/PD target was evaluated at both first TDM assessment (early aggressive PK/PD target attainment) and by taking into account the overall TDM assessments (overall aggressive PK/PD target attainment). Specifically, in patients who underwent more than one real-time TDM-guided ECPA, the average cefiderocol *f*C_ss_ was calculated by performing the mean of all observed *f*C_ss_ values (including both cefiderocol concentrations at first TDM assessment before any eventual dosing adjustment and the subsequent ones after eventual dosing adjustments), as previously performed [51].

### 4.5. Statistical Analysis

Demographics and clinical features of included patients were summarized as median and IQR for continuous variables, and as absolute values and percentages for categorical variables. Univariate and multivariate analyses were carried out for testing variables potentially associated with microbiological and clinical failure. In univariate analyses, categorical variables were compared by means of Fisher’s exact test or the chi-squared test where appropriate, whereas continuous variables were compared by means of the Mann–Whitney U test. All the independent covariates being associated with the univariate analysis with a *p*-value < 0.10 were included in an exploratory multivariate logistic regression model. Adjustment for age and sex was also performed for minimizing the risk of confounding factors. Statistical significance was defined as the occurrence of a *p*-value < 0.05. Statistical analysis was performed by means of MedCalc for Windows (MedCalc statistical software, version 19.6.1, MedCalc Software Ltd., Ostend, Belgium).

## Figures and Tables

**Figure 1:**
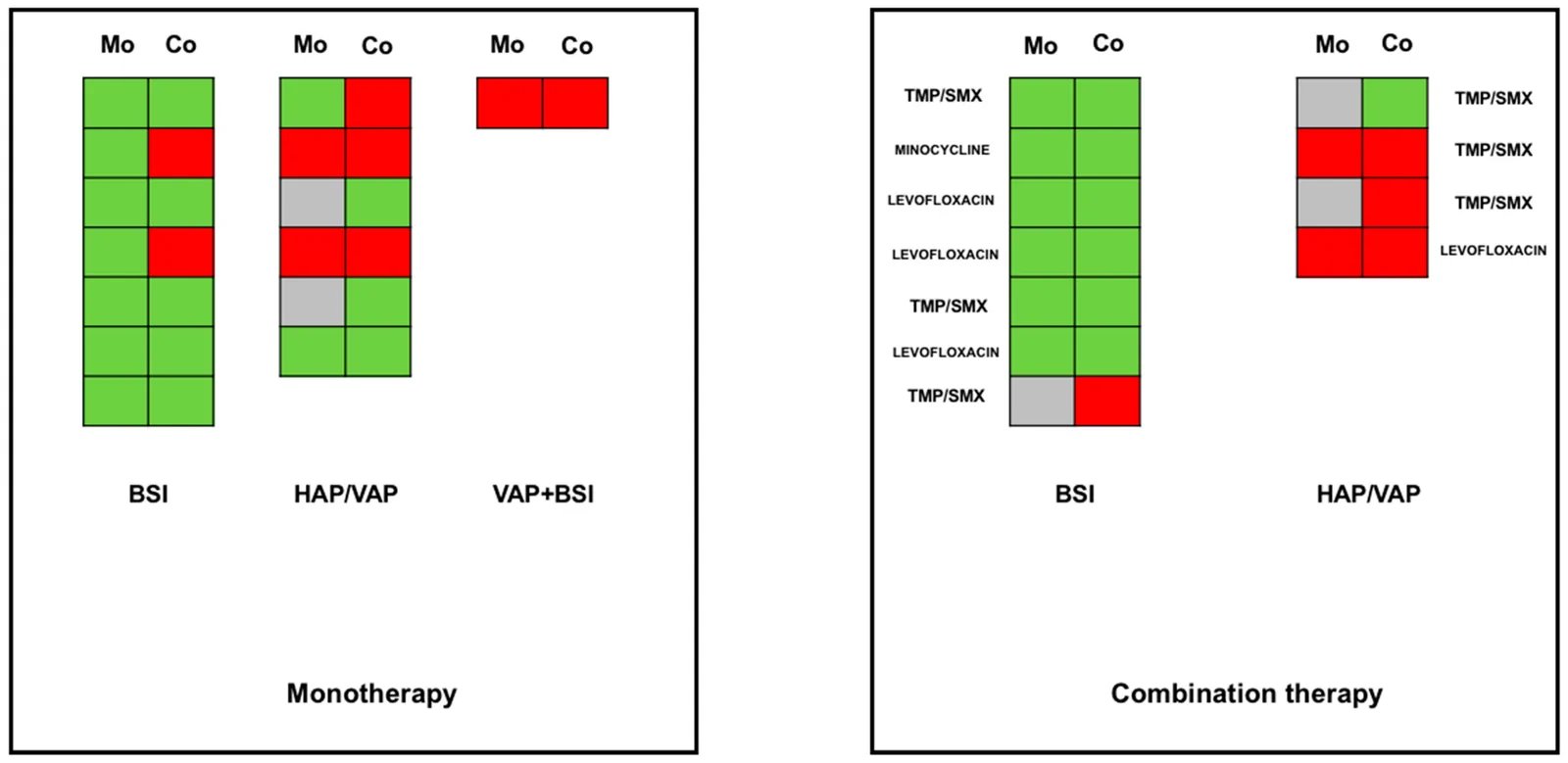
Summary of microbiological and clinical outcomes according to the site of infection and to the implementation of mono- or combination therapy. Each cell represents a single patient. All patients attained aggressive PK/PD target of cefiderocol. Green box: microbiological eradication/clinical cure; red box: microbiological/clinical failure; gray box: outcome not assessable due to unavailable follow-up microbiological cultures. BSI: bloodstream infections; Co: clinical outcome; HAP: hospital-acquired pneumonia; Mo: microbiological outcome; TMP/SMX: cotrimoxazole; VAP: ventilator-associated pneumonia.

**Table 1:** Demographics and clinical features of patients receiving targeted therapy with CI cefiderocol for treating documented infections caused by *Stenotrophomonas maltophilia*.

Variables	Overall Patients (n = 25)
*Demographics*	
Age (yrs; median; [IQR])	63.0 (40.0–67.0)
Gender (male/female; n [%])	18/7 (72.0/28.0)
Weight (kg; median; [IQR])	70.0 (60.0–75.0)
Body mass index (kg/m^2^; median; [IQR])	23.5 (21.6–24.8)
Obesity (n; [%])	0 (0.0)
*Underlying disease* (n; [%])	
Hematological malignancies	11 (44.0)
Solid organ transplant	6 (24.0)
Solid cancer	3 (12.0)
HIV infection	2 (8.0)
Others *	3 (12.0)
Immunosuppression	22 (88.0)
Charlson Comorbidity Index (median; [IQR])	5 (3–7)
*Setting* (n; [%])	
Intensive care unit	15 (60.0)
Hematology	5 (20.0)
Medicine	5 (20.0)
*Pathophysiological conditions*	
Vasopressors (n; [%])	10 (40.0)
Mechanical ventilation (n; [%])	9 (36.0)
Continuous renal replacement therapy (n; [%])	7 (28.0)
Hemoadsorptive filter (n; [%])	1 (4.0)
Augmented renal clearance (n; [%])	1 (4.0)
Baseline CL_CR_ (mL/min/1.73 m^2^; median; [IQR])	81.0 (25.0–107.0)
Serum albumin (g/dL; median; [IQR])	2.92 (2.76–3.40)
Hypoalbuminemia (n: [%])	19 (76.0)
*Site of infection* (n; [%])	
HAP/VAP	10 (40.0)
CR-BSI	9 (36.0)
BSI **	5 (20.0)
VAP+BSI	1 (4.0)
*Cefiderocol treatment regimens and PK/PD target attainment*	
Cefiderocol MD (g/die; median; [IQR])	6 (3–6)
Treatment duration (days; median; [IQR])	8 (5–14)
Combination therapy *** (n; [%])	11 (44.0)
Effective source control **** (n; [%])	10/12 (83.3)
MIC value (mg/L; median; [IQR])	0.125 (0.06–2)
Cefiderocol *f*C_ss_ (mg/L; median; [IQR])	22.8 (17.7–33.0)
Cefiderocol *f*C_ss_/MIC ratio (median; [IQR])	21.6 (12.2–87.3)
Early aggressive PK/PD target attainment (n; [%])	25 (100.0)
Overall aggressive PK/PD target attainment (n; [%])	25 (100.0)
Overall TDM-guided ECPA	48
TDM-guided ECPA per patient (median; [IQR])	1 (1–3)
Within desired range (ECPA; n; [%])	20 (41.7)
Below desired range (ECPA; n; [%])	1 (2.1)
Above desired range (ECPA; n; [%])	27 (56.2)
*Outcome* (n; [%])	
Clinical cure	15 (60.0)
Microbiological eradication *****	15/20 (75.0)
90-day resistance development ^#^	0/14 (0.0)
30-day mortality rate	10 (40.0)

BSI: bloodstream infection; CL_CR_: creatinine clearance; CR-BSI: catheter-related bloodstream infection; ECPA: expert clinical pharmacological advice; *f*C_ss_: free steady-state concentrations; HAP: hospital-acquired pneumonia; IQR: interquartile range; MD: maintenance dosing; MIC: minimum inhibitory concentration; PK/PD: pharmacokinetic/pharmacodynamic; TDM: therapeutic drug monitoring; VAP: ventilator-associated pneumonia. * Other underlying diseases included cardiocirculatory arrest (n = 1); metformin-associated lactic acidosis (n = 1), and hepatic cirrhosis (n = 1). ** Primary and secondary BSI were reported in 2 and 3 cases (having a possible abdominal source in 2 cases and cutaneous source in the other one), respectively. *** With cotrimoxazole (n = 6), levofloxacin (n = 4) or minocycline (n = 1). **** Source control was unfeasible in 13 cases. ***** Follow-up microbiological cultures were not performed in 5 cases. ^#^ Assessable only in the 14 cases having evaluable microbiological outcome and alive at 90 day.

**Table 2:** Univariate and multivariate analyses assessing potential predictors of microbiological failure in patients having available follow-up microbiological cultures.

Variables	Microbiological Eradication (n = 15)	Microbiological Failure (n = 5)	Univariate *p*-Value	Multivariate *p*-Value (OR; 95%CI)
*Demographics*				
Age (yrs; median; [IQR])	64.0 (37.5–69.0)	54.0 (51.0–64.0)	0.86	
Gender (male/female; n [%])	10/5 (66.7/33.3)	4/1 (80.0/20.0)	0.99	
Weight (kg; median; [IQR])	70.0 (60.5–74.5)	72.0 (65.0–77.0)	0.51	
Body mass index (kg/m^2^; median; [IQR])	24.2 (21.8–24.7)	22.5 (22.2–26.6)	0.93	
Obesity (n; [%])	0 (0.0)	0 (0.0)	0.99	
*Underlying disease* (n; [%])				
Hematological malignancies	7 (46.7)	1 (20.0)	0.60	
Solid organ transplant recipient	2 (13.3)	2 (40.0)	0.25	
Solid cancer	2 (13.3)	1 (20.0)	0.99	
HIV infection	1 (6.7)	1 (20.0)	0.45	
Others	3 (20.0)	0 (0.0)	0.54	
Immunosuppression	12 (80.0)	5 (100.0)	0.54	
Charlson Comorbidity Index (median; [IQR])	6 (3–7)	4 (4–10)	0.66	
*Setting* (n; [%])				
Intensive care unit	8 (53.3)	4 (80.0)	0.60	
Hematology	3 (20.0)	1 (20.0)	0.99	
Medicine	4 (26.7)	0 (0.0)	0.53	
*Pathophysiological conditions*				
Vasopressors (n; [%])	4 (26.7)	4 (80.0)	0.11	
Mechanical ventilation (n; [%])	3 (20.0)	4 (80.0)	**0.03**	
Continuous renal replacement therapy (n; [%])	3 (20.0)	3 (60.0)	0.13	
Hemoadsorptive filters (n; [%])	0 (0.0)	1 (20.0)	0.25	
Augmented renal clearance (n; [%])	1 (6.7)	0 (0.0)	0.99	
Baseline CL_CR_ (mL/min/1.73 m^2^; median; [IQR])	88.0 (29.3–105.0)	104.0 (60.0–118.5)	0.77	
*Site of infection* (n; [%])				
HAP/VAP	2 (13.3)	4 (80.0)	**0.013**	
CR-BSI	8 (53.3)	0 (0.0)	**0.05**	
BSI	5 (33.3)	0 (0.0)	0.27	
VAP+BSI	0 (0.0)	1 (20.0)	0.25	
Overall bacteremia	13 (86.7)	1 (20.0)	**0.013**	**0.016** **(OR 0.04;** **95%CI 0.01–0.54)**
Overall pneumonia	2 (13.3)	5 (100.0)	**0.001**	
*Cefiderocol treatment regimens and PK/PD target attainment*				
Treatment duration (days; median; [IQR])	7 (4–12)	13 (12–14)	0.11	
Combination therapy (n; [%])	6 (40.0)	2 (40.0)	0.99	
Effective source control (n; [%])	8/10 (80.0)	1/1 (100.0)	0.99	
Early aggressive PK/PD target attainment (n; [%])	15 (100.0)	5 (100.0)	0.99	
Overall aggressive PK/PD target attainment (n; [%])	15 (100.0)	5 (100.0)	0.99	

BSI: bloodstream infection; CI: confidence interval; CL_CR_: creatinine clearance; CR-BSI: catheter-related bloodstream infection; HAP: hospital-acquired pneumonia; IQR: interquartile range; OR: odds ratio; PK/PD: pharmacokinetic/pharmacodynamic; VAP: ventilator-associated pneumonia.

**Table 3:** Univariate and multivariate analysis assessing potential predictors of clinical failure.

Variables	Clinical Cure (n = 15)	Clinical Failure (n = 10)	Univariate *p*-Value	Multivariate *p*-Value (OR; 95%CI)
*Demographics*				
Age (yrs; median; [IQR])	63.0 (26.0–66.0)	63.0 (55.25–70.0)	0.45	
Gender (male/female; n [%])	9/6 (60.0/40.0)	9/1 (90.0/10.0)	0.18	
Weight (kg; median; [IQR])	70.0 (60.0–72.5)	71.0 (65.75–83.0)	0.28	
Body mass index (kg/m^2^; median; [IQR])	22.7 (20.9–24.5)	23.9 (22.3–26.2)	0.49	
Obesity (n; [%])	0 (0.0)	0 (0.0)	0.99	
*Underlying disease* (n; [%])				
Hematological malignancies	8 (53.3)	3 (30.0)	0.41	
Solid organ transplant recipient	2 (13.3)	4 (40.0)	0.18	
Solid cancer	2 (13.3)	1 (10.0)	0.99	
HIV infection	1 (6.7)	1 (10.0)	0.99	
Others	2 (13.3)	1 (10.0)	0.99	
Immunosuppression	13 (86.7)	9 (90.0)	0.99	
Charlson Comorbidity Index (median; [IQR])	5 (2.5–7.5)	5 (4–6.75)	0.54	
*Setting* (n; [%])				
Intensive care unit	7 (46.7)	8 (80.0)	0.21	
Hematology	3 (20.0)	2 (20.0)	0.99	
Medicine	5 (33.3)	0 (0.0)	0.06	
Vasopressors (n; [%])	4 (26.7)	6 (60.0)	0.12	
*Pathophysiological conditions*				
Mechanical ventilation (n; [%])	2 (13.3)	7 (70.0)	**0.009**	**0.008** **(OR 15.17;** **95%CI 2.03–111.35)**
Continuous renal replacement therapy (n; [%])	4 (26.7)	3 (30.0)	0.99	
Hemoadsorptive filters (n; [%])	0 (0.0)	1 (10.0)	0.40	
Augmented renal clearance (n; [%])	1 (6.7)	0 (0.0)	0.99	
Baseline CL_CR_ (mL/min/1.73 m^2^; median; [IQR])	76.0 (25.0–99.0)	100.0 (31.5–112.0)	0.44	
*Site of infection* (n; [%])				
HAP/VAP	4 (26.7)	6 (60.0)	0.12	
CR-BSI	7 (46.7)	2 (20.0)	0.22	
BSI	4 (26.7)	1 (10.0)	0.61	
VAP+BSI	0 (0.0)	1 (20.0)	0.40	
Overall bacteremia	11 (73.3)	4 (40.0)	0.12	
Overall pneumonia	4 (26.7)	7 (70.0)	**0.049**	
*Cefiderocol treatment regimens and PK/PD target attainment*				
Treatment duration (days; median; [IQR])	8.0 (6.0–12.5)	10.5 (4.75–13.75)	0.80	
Combination therapy (n; [%])	7 (46.7)	4 (40.0)	0.99	
Effective source control (n; [%])	7/8 (87.5)	3/4 (75.0)	0.99	
Early aggressive PK/PD target attainment (n; [%])	15 (100.0)	10 (100.0)	0.99	
Overall aggressive PK/PD target attainment (n; [%])	15 (100.0)	10 (100.0)	0.99	

BSI: bloodstream infection; CI: confidence interval; CL_CR_: creatinine clearance; CR-BSI: catheter-related bloodstream infection; HAP: hospital-acquired pneumonia; IQR: interquartile range; OR: odds ratio; PK/PD: pharmacokinetic/pharmacodynamic; VAP: ventilator-associated pneumonia.

## Data Availability

The data presented in this study are available upon request from the corresponding author. The data are not publicly available due to privacy restrictions.
